# Factors influencing the self-management ability among older adults experiencing intrinsic capacity decline: a cross-sectional study

**DOI:** 10.3389/fnagi.2024.1456167

**Published:** 2024-11-22

**Authors:** Qingcai Liu, Xiaoyang Li, Mingyue Hu, Yinan Zhao, Shuang Wu, Hui Feng

**Affiliations:** ^1^College of Humanities and Management, Hunan University of Chinese Medicine, Changsha, Hunan, China; ^2^Xiangya School of Nursing, Central South University, Changsha, Hunan, China

**Keywords:** intrinsic capacity, self-management ability, social support, older adults, influence factor

## Abstract

**Aims:**

This study investigated the current status of intrinsic capacity and self-management abilities and analyzed the factors influencing the self-management abilities of older adults experiencing intrinsic capacity decline.

**Methods:**

We included a sample of 382 older adults, with an average age of 72.92 ± 6.81 years, exhibiting intrinsic capacity decline in 38 communities in China. Questionnaires were administered using the sociodemographic questionnaire, the intrinsic capacity questionnaire, the social support scale, the self-rated health item, the eHealth literacy scale, and the self-management ability scale. The data were analyzed using descriptive statistics, Pearson correlation coefficients, and linear regression analysis.

**Results:**

In this study, 43.5% of older people had impairments in three of the five dimensions of intrinsic capacity, the most significant proportion. The self-management ability score of older adults experiencing intrinsic capacity decline was 67.05 ± 12.53 out of 100. There were significant associations between age, perceived social support, and intrinsic ability composite score with self-management abilities (Age: *β* = −0.263; *p* < 0.001; social support: *β* = 0.291; *p* < 0.001; intrinsic capacity composite score: *β* = 0.179; *p* < 0.001). Higher levels of self-management ability were associated with more effective maintenance of psychological capacity, regardless of the type of older adults experiencing IC decline (all *p* < 0.05). For example, among older adults experiencing cognitive decline, maintaining psychological function was positively associated with self-management abilities (*β* = 0.294; *p* < 0.01).

**Conclusion:**

The highest prevalence of concurrent impairments across three dimensions of intrinsic capacity was observed among older adults experiencing diminished intrinsic capacity. Fostering self-management abilities through social support and mental health interventions may benefit people experiencing intrinsic capacity decline.

## 1 Introduction

As aging progresses, there is an ongoing decline in physical, social, and psychological abilities. Therefore, it becomes crucial to focus on the factors that support healthy aging and maintain the aging populations’ well-being (Steptoe et al., 2015). The World Health Organization (WHO) asserts that the fundamental approach to promoting healthy aging lies in enhancing and preserving the intrinsic capacity of older individuals (World Health Organization, 2015, 2017). Intrinsic capacity (IC) refers to the set of all the physical and mental capabilities, primarily categorized into five key dimensions: cognition, vitality (i.e., homeostatic regulation, or balance between energy intake and energy utilization), locomotion (including muscular function), psychological (including mood and sociality), and sensory domain (including vision and hearing) (Belloni and Cesari, 2019).

Studies revealed high prevalence rates of IC decline among older adults, leading to higher levels of disability, hospitalization, and mortality (Prince et al., 2021; Zeng et al., 2021; Liu et al., 2021; Rarajam Rao et al., 2023). For instance, a systematic review revealed that 73.7% of older Chinese adults experience a decline in IC (Liu et al., 2023). Similar findings were reported in a cross-sectional study of community-dwelling older adults in Hong Kong, China, where 72.7% of participants had impairments in at least one IC domain (Leung et al., 2022). Likewise, a study in France involving 755 older individuals with an average age of 80.9 ± 7.3 years identified 699 participants with IC impairments (Tavassoli et al., 2021). Each subsequent IC decline had a direct correlation with a higher likelihood of developing frailty, instrumental activities of daily living (IADL) impairment, and activities of daily living (ADL) disability. Over 5 years, the likelihood of developing frailty rose by 47%, IADL impairment by 27%, and ADL disability by 23% (González-Bautista et al., 2021). Furthermore, a study demonstrated that the decline of one or more IC domains strongly and separately predicted the occurrence of dependency (pooled adjusted sub-hazard ratio 1.91, 95% confidence interval, CI 1.69–2.17) and mortality (pooled adjusted hazard ratio 1.66, 95% CI 1.49–1.85) (Prince et al., 2021). These findings highlight the significance of early preventive measures to maintain IC throughout aging.

Maintaining self-management abilities was critical for older adults’ health and independence, particularly as IC declines. Self-management mediated the relationship between quality of life and physical function (Ziv et al., 2024), and the factors influencing these abilities varied based on self-rated health status and social support (Suglo and Evans, 2020; van Schie et al., 2016). For example, van Schie et al. (2016) found that self-rated health positively correlated with effective self-management in individuals with schizophrenia. Similarly, a systematic review conducted in China demonstrated that individual and social factors impacted diabetic self-management (Luo et al., 2015). Within the diabetic population, a qualitative systematic review of 16 studies revealed that family support significantly enhanced their ability to control their diabetes (Suglo and Evans, 2020).

Health literacy, digital engagement, and eHealth literacy also influenced self-management among older adults (Geboers et al., 2016; Scheffer et al., 2021; Wong et al., 2022). For instance, Geboers et al. (2016) discovered a correlation between a lower health literacy score and inadequate abilities to manage oneself in those aged 75 and above. Furthermore, older adults with chronic conditions with self-management skills were likelier to engage in digital activities, such as emailing and online socialization (Scheffer et al., 2021). Nevertheless, determinants of self-management for older adults experiencing IC decline remain underexplored, highlighting a crucial research gap.

Notably, the factors affecting the self-management ability of older adults with different dimensions of IC decline may differ. For instance, factors such as age and education moderated the effects of perceived understanding on self-reported self-management behaviors among individuals with mild cognitive impairment (Kim et al., 2022). Similarly, among those with sensory impairments, self-management was influenced by prior access to healthcare, self-efficacy, age, and gender (Convery et al., 2019). Most existing studies have focused on disease-specific self-management, with fewer examining functional declines at an early stage. As an integrated measure of physical and mental functions, IC predicted subsequent care dependence better than examining diseases alone (George et al., 2021). Therefore, it is crucial to assess IC holistically to understand its influence on functional capacity.

Research indicated that maintaining positive mental attributes, such as resilience, adaptability, and motivation, could mitigate the impact of cognitive decline on self-management. A study showed that older adults with early dementia who participated in psychoeducational groups improved self-efficacy after 3 months compared to standard care participants (Cohen’s *d* = 0.35) (Quinn et al., 2016). Moreover, research showed that executive or subjective cognitive function affected daily self-management behaviors in older adults, highlighting the importance of cognitive health in supporting self-management (Kim et al., 2023). Additional studies suggested that enhancing sensory function in those with mild-to-moderate dementia may also boost self-efficacy (Leroi et al., 2020). Based on these findings, we hypothesized that declines in specific IC domains exert differential effects on self-management abilities, underscoring the complex interactions within these dimensions.

In summary, although research has identified factors such as social support, self-assessed health, and eHealth literacy as influential for self-management in individuals with specific diseases, there was a significant gap in understanding factors affecting self-management in older adults with IC decline. Due to the complexity of IC, this study first analyzed the current status of IC and then assessed the self-management abilities of older individuals experiencing IC decline. We hypothesized that sociodemographic factors, perceived social support, IC composite scores, self-rated health status, and eHealth literacy would be positively associated with self-management abilities in this population. Additionally, we categorized and investigated the factors influencing self-management abilities among older adults based on the different dimensions of IC.

## 2 Methods

### 2.1 Study design and settings

We employed the convenience sample method to conduct a cross-sectional study between August 1, 2021, and September 30, 2021. Our study recruited targeted individuals residing in 38 communities located in Changsha, China. Our research protocol was approved by the Behavioral Medicine and Nursing Ethics Committee of Xiangya School of Nursing, Central South University (No. E2021109). This study adhered to the Strengthening the Reporting of Observational Studies in Epidemiology (STROBE) guidelines for cross-sectional studies (von Elm et al., 2014).

The sample size calculation in this study was performed using the formula for estimating the mean parameter of a single sample size, as depicted in Equation 1. The *δ* represented the permissible error, i.e., the precision of the measurement, the *σ* represented the standard deviation (SD) of the metric within the population, and the *α* was the probability of committing a class of error, which was generally taken to be 0.05 bilaterally, i.e., $Z_{1-\alpha /2}=1.96$ .

(1) $$n={\left(\frac{Z_{1-\alpha /2}\sigma }{\delta }\right)}^{2}$$

The SD was expected to be 25, and the tolerable error was 3. Based on the formula, the sample size was calculated to be 267, and considering the 30% refusal rate, the sample size for this study was 382. Three hundred eighty-two older adults consented to participate and completed every data collection questionnaire.

### 2.2 Participants

Residents who lived or resided in communities in Changsha City, Hunan Province, China, were selected based on inclusion criteria: (1) aged ≥60 years; (2) had at least one dimension of functional decline, according to the assessment of the IC screening tools recommended by the World Health Organization (2017). We employed the Mini-Mental State Examination (MMSE) to assess cognitive functioning dimension, the Short Physical Performance Battery (SPPB) to assess mobility and physical functioning dimensions, the Mini Nutritional Assessment Short-Form (MNA-SF) to assess vitality dimensions, the Patient Health Questionnaire-9 (PHQ-9) to assess mental functioning dimensions, and the single sentence questioning of vision and Hearing Handicap Inventory for the Elderly–Screening version (HHIE-S) to assess sensory dimensions. The assessment criteria for each dimension of diminished function are detailed in the measures section; (3) had been a resident in one of the survey areas for a minimum of 6 months, and (4) provided informed consent and willingness to participate in the research.

Exclusion criteria for older adults included the presence of significant major diseases such as cancer, severe cardiovascular and cerebrovascular diseases, and the need for major organ transplants. Major diseases were identified through self-reporting and review of medical records. Additionally, individuals with severe impairment in any of the five dimensions of IC, such as an MMSE score of 9 or lower in the cognitive domain, a PHQ-9 score of 10 or higher in the psychological domain, an SPPB score of 7 or lower in the mobility domain, or an MNA-SF score of 8 or lower in the vitality domain, were also excluded. Furthermore, individuals with an HHIE-S score of 24 or higher or severe visual impairment in the sensory domain, those experiencing mental disorders, and those participating in other health-related intervention pilot studies were also excluded. Such impairments could limit the ability to perform self-management tasks, potentially introducing bias if participants faced excessive difficulty or required substantial assistance. Before participating in the study, all participants must complete and sign the informed consent form.

### 2.3 Measures

#### 2.3.1 Sociodemographic variables

The collected demographic characteristics included participants’ gender (male/female), age (years), education level (primary school and below/above primary school), current marital status (married/other), average monthly personal income (<2000 yuan/2000–4,000 yuan/>4,000 yuan), and Charlson Comorbidity Index (CCI). The CCI served as a tool for measuring the presence of comorbidities in patients. The index was derived from 17 specific disorders, such as cerebrovascular disease, chronic lung disease, congestive heart failure, malignant neoplasms, and AIDS. The scale spanned from 0 to 37 points. A higher CCI score indicated that patients had more significant and severe comorbidities (Charlson et al., 1987). The study classified the CCI into two categories: absence of comorbidity and presence of at least one condition.

#### 2.3.2 Intrinsic capacities levels

The evaluation focused on five dimensions of IC associated with the concepts and procedures outlined in the current WHO Guidelines on Integrated Care for Older People (ICOPE) regarding comprehensive assessment (WHO Guidelines Approved by the Guidelines Review Committee, 2017; Cesari et al., 2018). We established a threshold for each domain to determine if an individual had maintained their capacity or undergone a decline in IC. In keeping with prior research, IC decline was used to describe any capacity decrease (Prince et al., 2021). Declines in each domain of IC were assigned a value of 0, whereas all other cases were assigned a value of 1. The study utilized a composite score for the IC, which comprised the sum of individual components and ranged from 0 to 4. A higher IC composite score signified a greater degree of preserved functional capacity.

##### 2.3.2.1 Cognitive capacity

Cognitive function was assessed using the MMSE (Folstein et al., 1975). The MMSE measured cognitive function via interviews, in which interviewees were asked questions about time and space orientation, short-term memory, comprehension, and other cognitive dimensions. Scores ranged from 0 to 30, with higher scores indicating higher levels of cognitive function. The diagnostic boundary value was usually adjusted according to the level of education, such as college ≤26 points, middle school ≤24 points, primary school ≤23 points, and illiterate ≤22 points, indicating the decline of cognitive function (Tangalos et al., 1996).

##### 2.3.2.2 Locomotor capacity

Locomotor capacity was assessed using the SPPB (Guralnik et al., 1994). The SPPB was a functional test that measured gait speed (4 m walk), standing balance, and lower extremity strength and endurance (chair rise task). During the 4-meter walk, participants were asked to walk at their normal comfortable pace over a flat. The average of two trials was used. Three different static positions were evaluated for standing balance: feet side by side, semi-tandem (side of the heel of 1 foot touching the big toe of the other), and full tandem (heel of 1 foot in front of and touching the toes of the other foot). Participants were asked to try to hold each position for 10 s. For the chair rise task, participants were asked to stand up and sit down five times in a row as quickly as possible. Each test was scored on a scale of 0–4 points, with a summary performance score range of 0–12. If the participant could not perform a specific test, a score of 0 was assigned. The cumulative score for each measure was counted to obtain the total SPPB score (Guralnik et al., 1994). Those who scored ≥9 points were considered to have locomotor capacity.

##### 2.3.2.3 Vitality

Nutrition was assessed using the MNA-SF (Rubenstein et al., 2001). The MNA-SF included seven items: the degree of diet reduction and weight loss in the last 3 months, mobility, stress/acute disease, mental illness, and Body Mass Index (BMI) in the recent 3 months, as well as the calf circumference as an alternative item of BMI. The score varied from 0 to 3, with a total score of 0 to 14, positively correlated with nutrition. A score above 12 indicates normal nutritional status, a score of 8–11 indicates malnutrition risk, and a score of 0–7 indicates malnutrition. Nutrition was considered adequate for those who scored ≥12 points, with scores below this value identifying malnutrition.

##### 2.3.2.4 Psychological capacity

Psychological capacity was assessed using the PHQ-9. The PHQ-9 included nine items based on the Diagnostic and Statistical Manual of Mental Disorder-IV (DSM-IV) symptoms of depression (Kroenke and Spitzer, 2002). The investigators asked participants to specify how often they had been affected by depressed mood, lack of pleasure, despair, fatigue, loss of appetite, moving or speaking slowly, inattention, sleep difficulties, or suicidal thoughts during the past 2 weeks. The scoring standard was a 4-point system, ranging from 0 (none at all) to 3 (almost every day) (Kroenke and Spitzer, 2002). The total score was between 0 and 27. The higher the PHQ-9 score, the more serious the depression. In our study, a score of 5–9 was considered an indicator of subthreshold depression (Shrestha et al., 2021). Psychological capacity was considered adequate if those who scored <5 points, with scores below this value identifying psychological capacity decline.

##### 2.3.2.5 Sensory domain

Vision was assessed using a single-item custom question: “How would you rate your current vision (with glasses or contacts, if applicable)?” Vision was considered adequate when older people did not report “vision problems” that interfered with their activities, at least to some extent, and when they were not identified as functional blindness by the interviewer. The hearing was assessed using the HHIE-S. The HHIE-S was a series of 10 standardized questions designed to screen for self-assessed hearing handicaps in older individuals. The questions included five social or situational items and five emotional response items. A response of “yes” was given 4 points, “sometimes” was given 2 points, and “no” was given 0 points. Those who scored ≤7 points were considered to have a hearing capacity, with scores above this value identifying hearing loss (Ventry and Weinstein, 1983). If the respondent had vision loss or/and hearing impairment, the researchers adjudicated them as having reduced sensory function.

#### 2.3.3 Perceived social support

Perceived Social Support was assessed by the Perceived Social Support Scale (PSSS) (Zimet et al., 1988). This measure has been extensively used to measure perceived social support from three sources: family, friends, and significant others. This questionnaire, which involved 12 items, asked participants to rate on a 5-point Likert-type scale ranging from 1 (very strongly disagree) to 5 (very strongly agree). Higher scores represented higher levels of perceived social support.

#### 2.3.4 Self-rated health status

A single question assessed self-reported health, ‘What is your current health status?’. Participants were asked to rate on a 5-point Likert-type scale ranging from 1, indicating poor health, to 5, excellent health status.

#### 2.3.5 eHealth literacy

eHealth literacy was assessed using the Simplified Chinese eHealth Literacy Scale (Xu et al., 2020). People tested their level of agreement with each statement on a 5-point Likert scale from 1 strongly disagree to 5 strongly agree. The final score ranged from 8 to 40, and higher scores indicated higher eHealth literacy.

#### 2.3.6 Outcome variable

##### 2.3.6.1 Self-management ability

We measured self-management abilities with the Self-Management Ability Scale-30 (SMAS-30) (Schuurmans et al., 2005). This tool mainly evaluated the self-management ability of older adults to maintain well-being, which included six dimensions: taking the initiative, investment behavior, variety, multi-functionality, self-efficacy, and a positive frame of mind. SMAS-30 consisted of 30 items, scored by five and 6-point Likert scales. Overall, SMAS scores ranged from 0 to 100, and the mean of the sub-scale scores with higher scores indicated higher self-management ability.

### 2.4 Research process

#### 2.4.1 Personnel training

The survey was cooperatively done by a PhD student in nursing and two postgraduate students holding a Master of Nursing degree. Every investigator possessed specialized knowledge, abilities, and a responsible approach to conducting research. Before starting the survey, each investigator participated in professional knowledge training, which covered key topics such as the study’s objectives, questionnaire content, privacy protection, communication skills, and other relevant areas. The training was designed to ensure a consistent understanding among all investigators, enabling them to address any questions the older respondents raised effectively.

#### 2.4.2 Preliminary investigation

Before starting the study, six participants were selected based on the inclusion and exclusion criteria outlined in the study. These participants then underwent individual preliminary surveys to assess the reliability of the measurement tool and the rationality of the investigation process. From the level of language clarity and the ease of understanding, the questionnaire was in line with their language expression habits, made a record, in the case without changing the original intention, expressed in the native language, and formed the survey questionnaire’s final version.

#### 2.4.3 Recruitment

We utilized a dual recruitment approach for this study, combining promotional posters at community health service centers with direct recommendations from family physicians. The posters primarily encompass the research’s themes and objectives, the respondents’ requests, the study’s content and methods, the potential advantages of participant involvement, the ways of participation and contact, and information regarding the research institution or researcher. Although posters had the advantage of quickly attracting a significant number of older individuals, they may also attract a substantial number of participants who did not fit the specific criteria for inclusion. This could make the screening process more challenging.

We also relied on family physicians to assist with recruitment to address this. The community healthcare center in China provides complimentary medical assessments for people over 60. Family physicians had a strong connection with seniors and could promote their active participation in research (Manohar et al., 2019). Consequently, the family doctors at the healthcare institution aided us in recruiting. Family doctors advise the involvement of older people who may fulfill the eligibility requirements in our research after a thorough medical assessment. The researchers assessed the potential research participants that had been recruited and identified the subjects that fulfilled the requirements for inclusion.

#### 2.4.4 Collection and distribution of questionnaires

With the help of the community health center manager, the investigators conducted face-to-face interviews with older adults who volunteered to participate in this study. Initially, the investigator assessed the age of the ID card to determine if the possible study participants fulfilled the inclusion requirements. Subsequently, the investigator communicated with the older individuals to identify their willingness to participate in the survey.

**Figure 1:**
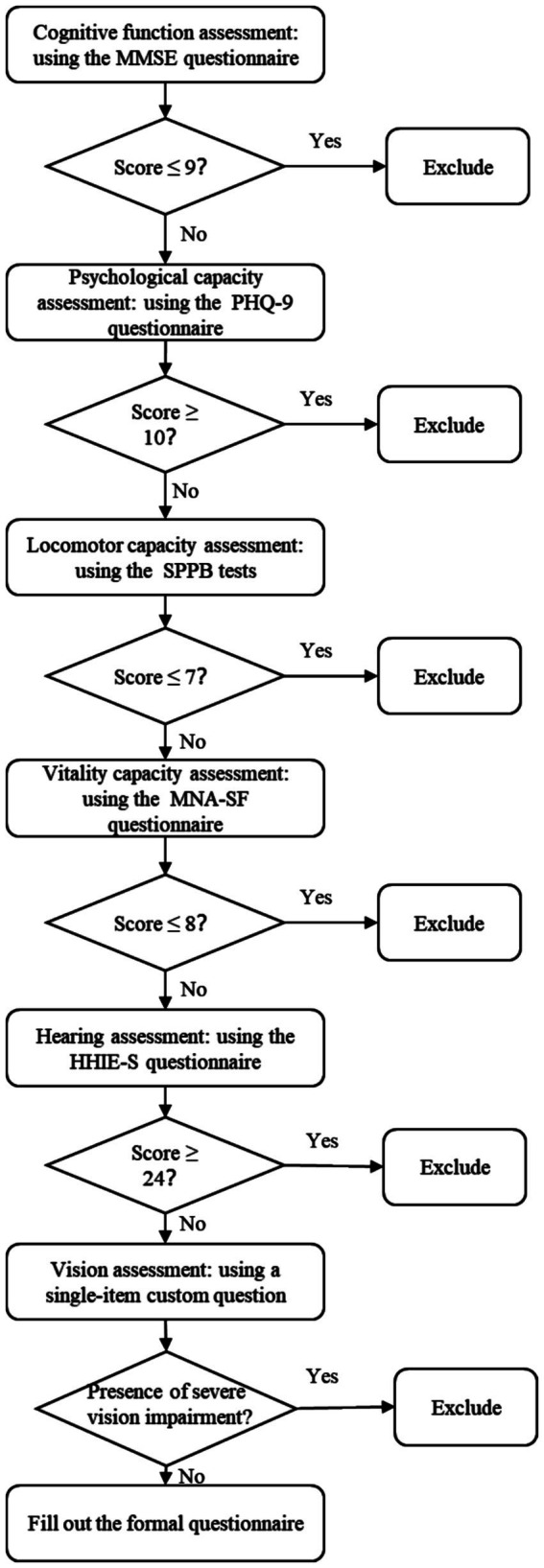
Flowchart of intrinsic ability screening. MMSE, Mini-Mental State Examination; PHQ-9, Patient Health Questionnaire-9; SPPB, Short Physical Performance Battery; MNA-SF, Mini Nutritional Assessment Short-Form; HHIE-S, Hearing Handicap Inventory for the Elderly–Screening version.

The investigator evaluated the five dimensions of IC using the Intrinsic Capacity Assessment Tool, explicitly focusing on individuals aged 60 years and above who expressed willingness to engage in the study. The investigator followed the process in Figure 1 to determine whether older adults had severe cognitive, psychological, mobility, vitality, and visual and hearing impairments. If older adults had severe IC impairments, they were excluded from continuing with the follow-up questionnaire; instead, the investigators thoroughly explained the purpose of the survey to the participants and emphasized privacy protection.

If the potential participants had learned all the relevant information, they would have completed the questionnaire after signing the informed consent form. The researchers read out the survey questions and marked the participants’ answers. The questionnaire took 20 min to complete. Upon completion, participants received a gift worth 50 yuan.

### 2.5 Statistical methods

Data were collected using Epidata 3.1 software (EpiData Association, Odense, Denmark). After data entry and checking, data analyses were performed with SPSS version 26.0. Before beginning formal analysis, we evaluated the normal distribution of each variable. The normality test, which depended on the skewness and kurtosis coefficients, stated that if the absolute value of kurtosis was below 10 and the absolute value of skewness was below 3, the data, while not perfectly regular, could be considered reasonably close to normal. The age variable had a skewness of 0.859 and a kurtosis of 0.033, measured in absolute terms. The skewness value for the variable representing the total social support score was 0.838, whereas the kurtosis value was 2.552. The eHealth literacy variable had a skewness of 0.818 and a kurtosis of 0.462, measured in absolute terms. The skewness of the self-management abilities variable was 0.379, while the kurtosis was 0.046. The data for the variables in this study could be considered to follow a normal distribution.

Continuous variables were described as mean ± SD, and binary and categorical variables were expressed as frequency (percentage). To assess differences in self-management scores across groups, we employed independent *t*-tests and one-way ANOVA for categorical independent variables, aligning with our aim to investigate whether sociodemographic factors influence self-management abilities.

Pearson’s correlation analysis explored the linear relationships between age, social support, eHealth literacy, and self-management abilities. This step was critical to address our hypothesis that psychosocial factors, such as social support and eHealth literacy, were associated with self-management abilities in older adults experiencing IC decline.

Multiple linear regression was then employed to assess how various sociodemographic variables, eHealth literacy, social support, self-rated health, and IC, predicted self-management abilities. To avoid multicollinearity, we calculated the variance inflation factor (VIF), with none of the variables exceeding the threshold of 10. We also checked for autocorrelation using the Durbin-Watson (D-W) test, where a value close to 2 indicated no autocorrelation, supporting the validity of our model.

Additionally, we ensured that residuals followed a normal distribution by plotting a standardized residual histogram and confirmed homoscedasticity through a scatter plot of standardized residuals against predicted values. These checks ensured that the data met the assumptions necessary for regression analysis. The level of statistical significance was set at 0.05 for all analyses.

## 3 Results

### 3.1 Demographic characteristics

**Figure 2:**
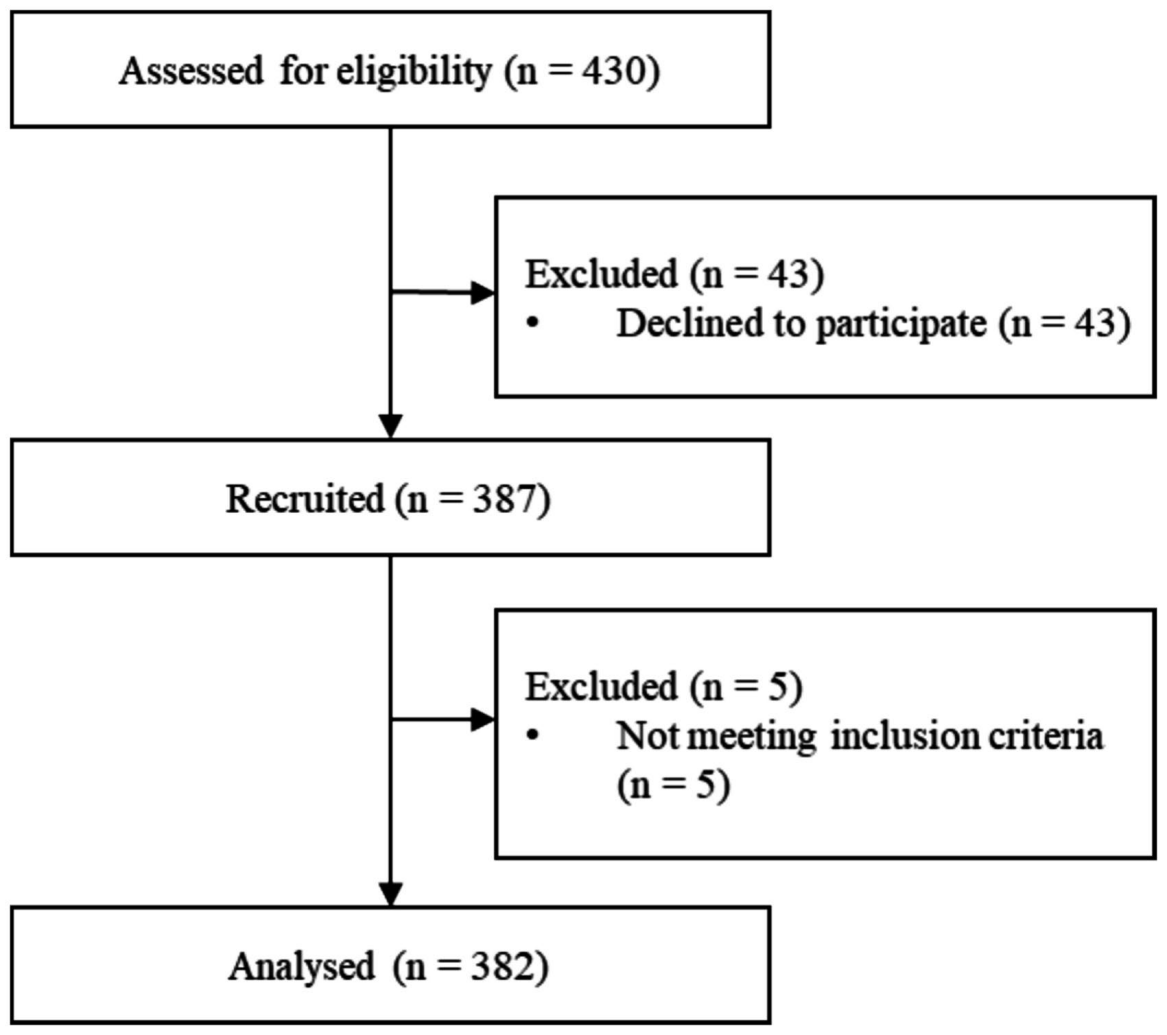
Flowchart of research subject recruitment.

**Figure 3:**
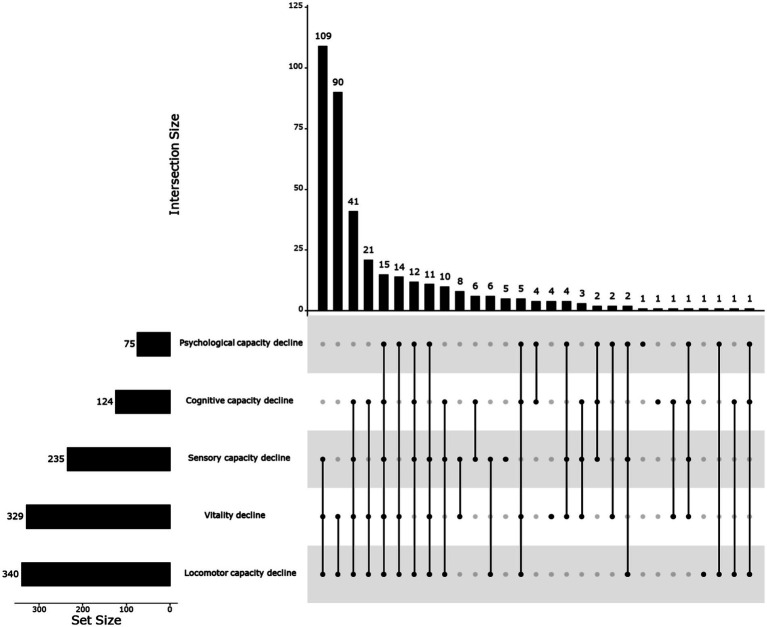
Frequency of combination of different dimensions of intrinsic capacity decline. Created in UpSetR (Lex et al., 2014).

Three hundred eighty-seven participants were recruited from 38 communities (see Figure 2). Five out of 387 participants were not eligible for inclusion and exclusion criteria. Finally, 382 older adults experiencing IC decline were included in the study. Out of the total number of older adults, 15 individuals (3.93%) experienced losses in all five sub-dimensions of IC. Additionally, 70 individuals (18.32%) had declines in 4 dimensions, 166 individuals (43.46%) had declines in 3 dimensions, 119 individuals (31.15%) had declines in 2 dimensions, and 12 individuals (3.14%) had declines in only one dimension. Out of the five areas of IC decline, 75 (19.63%) older individuals experienced a decline in their psychological capacity. Approximately 32.46% of the subjects experienced a decline in cognitive function. 89.01% of the subjects experienced a decrease in their locomotor capacity. Of all the participants, 329 (86.13%) reported decreased vitality. Out of the participants, 147 individuals (38.48%) who were older did not have either hearing loss or visual impairment. Figure 3 depicts the frequency of various combinations of IC decline aspects reported by the respondents. The bars represented the frequency of each combination. The dots linked to the lines beneath the bars indicate the dimension that indicates the reduction of IC. The most prevalent occurrence was a simultaneous decrease in the sensory, vitality, and locomotor domains, with a sample size of 109.

Male individuals accounted for around 52.09% of the sample. The average age was 72.92, with an SD of 6.81 years. 60.73% of the total respondents had finished primary school or lower. Most participants were married, accounting for 72.00% of the total. Approximately 60% of individuals possessed a monthly salary below 2,000 yuan. The study comprised 282 adults with a CCI score of 0, 93 individuals with a score of 1, and 7 individuals with a score of 2. No individual achieved a score of 3 or higher. For the subsequent analysis, older people with scores of 1 and 2 were combined into one group, while those with a score of 0 were classified separately. The average IC composite score was 2.94, with an SD of 1.04. The self-management score was 67.05, with an SD of 12.53. Table 1 provides a more comprehensive description of the background characteristics.

**Table 1:** Characteristics of the total sample (*n* = 382).

Variable	Category	Mean ± SD or *n* (%)
Gender	Male	199 (52.09)
	Female	183 (47.91)
Age		72.92 ± 6.81
Educational level	Primary school and below	232 (60.73)
	Above primary school	150 (39.27)
Marital status	Single/divorced/widowed	107 (28.00)
	Married	275 (72.00)
Income	<2000 yuan	232 (60.73)
	2000–4,000 yuan	98 (25.65)
	>4,000 yuan	52 (13.62)
Perceived social support		46.56 ± 8.11
Self-rated health status	Poor/Fair	213 (55.76)
	Good/very good/excellent	169 (44.24)
eHealth literacy		16.19 ± 9.44
Charlson Comorbidity Index	0	282 (73.82)
	≥1	100 (26.18)
Self-management score		67.05 ± 12.53

SD, standard deviation.

### 3.2 Relationship between different characteristics of older adults experiencing IC decline and self-management abilities

We utilized *t*-tests and one-way analysis of variance to assess the disparities in self-management ability scores among older adults experiencing decreases in IC, considering various demographic characteristics. The factors influencing self-management ability encompassed educational level, marital status, income, self-rated health, and IC (including psychological, cognitive, sensory, and vitality dimensions). Table 2 demonstrates that all covariates, except for gender and locomotor capacity, exhibited statistically significant associations with differences in self-management abilities scores.

**Table 2:** Comparison of self-management ability scores among older adults with intrinsic capacity decline with different characteristics.

Variable	Category	Mean ± SD	Statistic value	*p*-value
Gender			0.091^c^	0.928
	Male	67.11 ± 12.61		
	Female	66.99 ± 12.47		
Educational level			−5.268^c^	**<0.001**
	Primary school and below	64.49 ± 12.64		
	Above primary school	71.01 ± 11.30		
Marital status			−4.634^a^	**<0.001**
	Single/divorced/widowed	62.41 ± 14.88		
	Married	68.86 ± 10.99		
Income			10.535^b^	**<0.001**
	<2000 yuan	64.84 ± 12.55		
	2000–4,000 yuan	69.49 ± 11.29		
	>4,000 yuan	72.30 ± 12.46		
Self-rated health status			−2.724^a^	**0.007**
	Poor/fair	65.51 ± 13.16		
	Good/very good/excellent	69.00 ± 11.42		
Charlson Comorbidity Index			1.282^a^	0.201
	0	67.54 ± 11.69		
	≥1	65.67 ± 14.61		
Cognitive capacity			−8.042^a^	**<0.001**
	Cognitive capacity decline	60.17 ± 13.05		
	Normal cognitive capacity	70.36 ± 10.83		
Locomotor capacity			1.448^c^	0.153
	Locomotor capacity decline	67.34 ± 12.71		
	Normal locomotor capacity	64.72 ± 10.83		
Vitality			6.539^c^	**<0.001**
	Vitality decline	68.67 ± 11.85		
	Normal vitality	57.03 ± 12.06		
Psychological capacity			−7.623^a^	**<0.001**
	Psychological capacity decline	57.83 ± 13.65		
	Normal psychological capacity	69.30 ± 11.15		
Sensory capacity			−2.790^a^	**0.006**
	Sensory capacity decline	65.65 ± 13.29		
	Normal sensory capacity	69.29 ± 10.87		

^aThe statistical value is t-value; bThe statistical value is an F-value; cThe statistical value is t’ value; significances were bold.^

### 3.3 Correlation analysis of age, social support, eHealth literacy, and self-management abilities among older adults experiencing IC decline

The results presented in Table 3 indicated a negative correlation between age and eHealth literacy and self-management abilities among older adults experiencing IC decline. The correlation coefficient for eHealth literacy was −0.312 (*p* < 0.001), while for self-management abilities, it was −0.379 (*p* < 0.001). On the other hand, social support was positively correlated with self-management abilities, with a correlation coefficient of 0.392 (*p* < 0.001). Additionally, eHealth literacy positively correlated with self-management abilities, with a correlation coefficient of 0.243 (*p* < 0.001).

**Table 3:** Correlation analysis of age, social support, eHealth literacy, and self-management abilities among older adults with reduced intrinsic capacity (*n* = 382).

Variable	Age	Perceived social support	eHealth literacy	Self-management abilities
Age	1	–	–	–
Perceived social support	−0.072	1	–	–
eHealth literacy	**−0.312**	0.087	1	–
Self-management abilities	**−0.379**	**0.392**	**0.243**	1

Significances were in bold.

### 3.4 Multivariable linear regression analysis for the influencing factors of self-management abilities among older adults experiencing IC decline

As seen in Table 4, the results showed that age, gender, perceived social support, and IC composite score were significantly associated with self-management abilities. One unit increase in age was significantly associated with one unit decrease in self-management ability (*β* = −0.263; *p* < 0.001). One unit increase in perceived social support was significantly associated with one increase in self-management ability (*β* = 0.291; *p* < 0.001). One unit increase in the IC composite score was significantly associated with one unit increase in self-management ability (*β* = 0.179; *p* < 0.001).

**Table 4:** Influencing factors of self-management abilities using multiple linear regression analysis (*n* = 382).

Factors	Self-management ability	
	*β*	*p*-value
Gender (ref: male)	0.060	0.177
Age (ref: one unit increase)	**−0.272**	**<0.001**
Educational level (ref: primary school and below)	0.090	0.085
Marital status (ref: single/divorced/widowed)	0.068	0.156
Income (ref: <2000 yuan)		
2000–4,000 yuan	0.040	0.411
>4,000 yuan	0.058	0.277
Perceived social support	**0.296**	**<0.001**
Self-rated health status (ref: poor/fair)	0.061	0.162
eHealth literacy	0.052	0.281
Charlson Comorbidity Index (ref: no comorbidity)	0.009	0.843
IC composite score	**0.167**	**<0.001**

IC, intrinsic capacity; significances were in bold.

### 3.5 Multiple linear regression analyses for factors of self-management abilities across subgroups classified by dimensions of IC

The results showed that age was significantly associated with self-management abilities regardless of the dimension in which an older adult experienced a decline in IC (see Table 5). Regardless of whether other dimensions of IC of older adults were reduced, sensory function was not related to their self-management abilities (all *p* > 0.05). Psychological capacity was positively related to the self-management ability of older adults, with declines in the other four dimensions.

**Table 5:** Factors of self-management abilities across subgroups classified by dimensions of IC: multiple linear regression analysis (*n* = 382).

Variable	Self-management ability													
	Cognitive capacity decline (*n* = 124)^a^		Locomotor capacity decline (*n* = 340)^b^		Vitality decline (*n* = 329)^c^		Psychological capacity decline (*n* = 75)^d^		Sensory capacity decline (*n* = 235)^e^		Sensory capacity-vision loss (*n* = 174)^f^		Sensory capacity-hearing loss (*n* = 128)^g^	
	*β*	*p*-value	*β*	*p*-value	*β*	*p*-value	*β*	*p*-value	*β*	*p*-value	*β*	*p*-value	*β*	*p*-value
Gender (ref: male)	0.143	0.083	0.090	0.050	0.069	0.169	−0.099	0.316	0.067	0.225	0.083	0.190	0.012	0.875
Age (ref: one unit increase)	**−0.313**	**0.001**	**−0.174**	**0.002**	**−0.171**	**0.002**	**−0.449**	**<0.001**	**−0.210**	**0.002**	**−0.229**	**0.005**	**−0.237**	**0.009**
Educational level (ref: primary school and below)	**0.187**	**0.031**	0.065	0.228	0.051	0.405	0.121	0.321	0.094	0.125	**0.176**	**0.009**	−0.012	0.887
Marital status (ref: single/divorced/widowed)	0.133	0.122	0.084	0.090	0.080	0.128	0.005	0.963	**0.125**	**0.041**	0.127	0.076	0.121	0.151
Income (ref: <2000 yuan)	–	–	–	–	–	–	–	–	–	–	–	–	–	–
2000–4,000 yuan	0.039	0.624	0.058	0.251	0.068	0.226	0.130	0.227	0.036	0.522	0.042	0.509	0.017	0.818
>4,000 yuan	−0.048	0.545	0.072	0.193	0.101	0.103	−0.015	0.893	0.087	0.173	0.089	0.190	0.029	0.746
Perceived social support	0.029	0.726	**0.213**	**<0.001**	**0.248**	**<0.001**	**0.214**	**0.029**	**0.160**	**0.006**	0.084	0.185	**0.187**	**0.021**
Self-rated health status (ref: poor/fair)	0.111	0.138	0.010	0.818	0.031	0.542	−0.129	0.159	0.101	0.064	0.086	0.148	0.118	0.119
eHealth literacy	0.061	0.473	−0.015	0.763	−0.002	0.964	0.167	0.168	0.008	0.895	0.044	0.521	0.073	0.385
Charlson Comorbidity Index (ref: no comorbidity)	0.016	0.837	0.054	0.237	0.049	0.326	−0.023	0.813	0.050	0.358	0.027	0.659	0.051	0.499
Cognitive capacity (ref: cognitive capacity decline)	–	–	**0.178**	**<0.001**	**0.162**	**0.002**	0.071	0.546	**0.133**	**0.027**	**0.159**	**0.020**	0.107	0.205
Locomotor capacity (ref: locomotor capacity decline)	**0.204**	**0.011**	–	–	0.008	0.880	0.171	0.068	0.104	0.055	**0.117**	**0.048**	0.109	0.145
Vitality (ref: vitality decline)	−0.093	0.288	**−0.131**	**0.012**	–	–	0.023	0.836	−0.103	0.090	−0.025	0.722	−0.143	0.096
Psychological capacity (ref: psychological capacity decline)	**0.294**	**0.001**	**0.192**	**<0.001**	**0.160**	**0.002**	–	–	**0.228**	**<0.001**	**0.248**	**<0.001**	**0.192**	**0.017**
Sensory capacity (ref: sensory decline)	0.024	0.759	0.031	0.493	0.019	0.692	0.095	0.324	–	–	–	–	–	–
Sensory capacity-vision (ref: vision loss)	–	–	–	–	–	–	–	–	–	–	–	–	0.132	0.090
Sensory capacity-hearing (ref: hearing loss)	–	–	–	–	–	–	–	–	–	–	0.040	0.514	–	–

Significances were in bold. Sensory capacity decline means the presence of hearing or/and visual impairment. ^a^Results of multivariate analysis of factors affecting self-management ability among older adults with cognitive capacity decline (*n* = 124). ^b^Results of multivariate analysis among older adults with locomotor capacity decline (*n* = 340). ^c^Results of multivariate analysis among older adults with vitality capacity decline (*n* = 329). ^d^Results of multivariate analysis among older adults with psychological capacity decline (*n* = 75). ^e^Results of multivariate analysis among older adults with sensory capacity decline (*n* = 235). ^f^Results of multivariate analysis among older adults with vision loss (*n* = 174). ^g^Results of multivariate analysis among older adults with hearing loss (*n* = 128).

As to the cognitive capacity decline, a year increase in age (*β* = −0.313; *p* < 0.01) was associated with lower self-management abilities. The higher the education level, the higher the self-management ability of older adults with cognitive impairment compared to those with low levels of education (*β* = 0.187; *p* < 0.05). Locomotor and psychological capacities were positively related to self-management in older adults with cognitive decline (Locomotor: *β* = 0.204; *p* < 0.05; psychological: *β* = 0.294; *p* < 0.01).

Regarding the locomotor capacity decline, a year increase in age (*β* = −0.174; *p* < 0.01) was associated with lower overall self-management. One unit increase in the perceived social support (*β* = 0.213; *p* < 0.001) was associated with a higher overall self-management. Cognitive capacity and psychological capacities were positively related to self-management in older adults experiencing the locomotor capacity decline (Cognition: *β* = 0.178; *p* < 0.001; psychological: *β* = 0.192; *p* < 0.001). However, vitality was negatively related to self-management in older adults with the locomotor capacity decline (*β* = −0.131; *p* < 0.05).

As to the vitality decline, a year increase in age (*β* = −0.171; *p* < 0.01) was associated with a lower overall self-management. One unit increase in the perceived social support (*β* = 0.248; *p* < 0.001) was associated with a higher overall self-management. Cognitive and psychological capacities were positively related to self-management abilities in older adults with decreased vitality (Cognition: *β* = 0.162; *p* < 0.01; psychological: *β* = 0.160; *p* < 0.01).

As to the psychological capacity decline, a year increase in age (*β* = −0.449; *p* < 0.001) was associated with lower overall self-management. One unit increase in perceived social support (*β* = 0.214; *p* < 0.05) was associated with a higher overall self-management. None of the other four dimensions of IC was related to the self-management abilities of older adults with reduced psychological function.

Regarding the sensory capacity decline, age negatively predicts self-management ability (*β* = −0.210; *p* < 0.01). Marital status was associated with self-management in older adults with sensory decline (*β* = 0.125; *p* < 0.05). One unit increase in perceived social support (*β* = 0.160; *p* < 0.01) was associated with a higher overall self-management. Cognition and psychological capacities were positively related to self-management ability (Cognition: *β* = 0.133; *p* < 0.05; psychological: *β* = 0.228; *p* < 0.001).

As to the sensory capacity – vision loss, a year increase in age (*β* = −0.229; *p* < 0.01) was associated with a lower overall self-management. Education level, cognition, locomotor, and psychological capacities were positively related to self-management ability (Education: *β* = 0.176; *p* < 0.01; cognition: *β* = 0.159; *p* < 0.05; locomotor: *β* = 0.117; *p* < 0.05; psychological: *β* = 0.248; *p* < 0.001).

As to the sensory capacity – hearing loss, a year increase in age (*β* = −0.237; *p* < 0.01) was associated with a lower overall self-management. One unit increase in perceived social support (*β* = 0.187; *p* < 0.05) was associated with a higher overall self-management. The higher the self-management ability of those who maintain better psychological function compared to those who have reduced psychological function (*β* = 0.192; *p* < 0.05).

## 4 Discussion

Our study demonstrated that the decline in sensory, vitality, and locomotor functions at the same time was widespread among older adults experiencing IC decline. The results of this study showed that the self-management ability score of older adults experiencing IC decline was 67.05 ± 12.53 out of 100, which was mainly related to age, perceived social support, and IC composite scores. Interestingly, self-rated health, eHealth literacy, and self-management ability did not correlate in this population. Notably, regardless of the dimension in which an older adult experienced a decline in IC, age consistently influenced their self-management abilities. Similarly, social support remained a crucial factor affecting self-management abilities across all dimensions of IC decline in older adults, except for cognitive function. Furthermore, psychological function was consistently linked to self-management ability, irrespective of which IC dimension had decreased. Our findings revealed these relationships; however, the study’s cross-sectional nature limited causal conclusions, allowing only inferences of correlation and suggesting avenues for further investigation into the underlying mechanisms.

In examining the decline of different dimensions of IC, we discovered that the most common occurrence was the simultaneous decrease in sensory, vitality, and locomotor functions. These findings contrasted with previous studies, such as that by Jiang et al. (2023), which reported a greater prevalence of declines in sensory, cognitive, and locomotor functions among older adults with reduced IC. Furthermore, a study conducted in India involving 1,000 adults aged 60 and above found that the majority experienced a decline in only one specific aspect of their intrinsic capabilities, while only 91 individuals exhibited declines across all three dimensions (Rarajam Rao et al., 2023). The more significant proportion observed in our study may be attributed to the older age distribution of our individuals compared to those in the Indian study. Research has demonstrated that as individuals get older, the combination of functional impairments grows more intricate, and those with a more significant number of impairments are at a greater risk of mortality (Chen et al., 2023). This highlights the critical need for personalized early prevention, intervention, and care strategies tailored to the unique challenges faced by older adults experiencing declines in IC.

Our study also revealed a substantial correlation between age and self-management abilities in all IC declines, corroborating previous research that indicates older patients typically exhibit worse self-management capabilities than their younger counterparts (Scheffer et al., 2021; Lan et al., 2021; Bos-Touwen et al., 2015). This decline in self-management may stem from the deterioration of physical function due to aging or chronic illness. Consequently, healthcare professionals should prioritize enhancing the self-management abilities of older adults by providing tailored support in areas such as exercise, nutrition, illness management, and other interventions.

Furthermore, our study highlighted a correlation between perceived social support and self-management abilities. Based on the comprehensive conceptual framework of social support, it is believed that social support can enhance developmental outcomes by inducing various positive psychological changes, such as improvements in self-evaluation motivation and emotional well-being. Individuals could enhance their self-regulation and coping mechanisms through social support, such as managing emotions and thoughts and controlling behavior (Feeney and Collins, 2015). Similar to previous research on chronic illness, our study also found that social support influenced self-management (Lu et al., 2021). Positive support from family and friends, especially those nearby, helped with various self-management aspects, including providing medication reminders, preparing healthy food, and accompanying individuals to exercise (Lundberg and Thrakul, 2012). However, older adults also reported a lack of peer, family, and community support as a self-management barrier (Garnett et al., 2018). Our study found no correlation between social support and the self-management abilities of older people with cognitive impairments. This may be attributed to cognitive decline affecting their perception of social support and overall self-management capacity. Future studies should explore additional modifiable factors influencing self-management in older adults experiencing cognitive decline.

Additionally, we found that higher IC composite scores were associated with better self-management abilities. Previous systematic reviews had confirmed that health status, including physical and cognitive functioning, symptoms, and comorbidities, influenced self-management capacities (Schulman-Green et al., 2016). The reason might be that IC impairment added complexity to healthcare regimens and contributed to symptoms that interfered with self-management efforts. For example, one study found that perceived health, functional ability, and vitality were all significantly associated with self-management capacities: patients with lower self-management capacities scores reported worse perceived health, worse functional ability, and worse perceived vitality (Skolasky et al., 2011). Our study also highlighted an association between maintaining psychological functioning and improved self-management abilities, regardless of the type of older adults experiencing IC decline. Older adults who maintained better psychological functioning might have better psychological resilience. When they experience a diminished IC in a particular dimension, they might take the initiative to solve problems based on their usual learning, work, and life experiences or actively seek help from others to promote a level of self-management (Cui et al., 2023).

Interestingly, our study found that self-rated health was not an independent factor influencing self-management ability. At the same time, previous research has shown that perceived health status affects health self-management. One study found that in an education program for people experiencing type II diabetes, patients with optimal self-related health were significantly associated with increased self-management skills (Laursen et al., 2016). Another study found that self-rated health improved after a self-management skill program in people experiencing multiple chronic health conditions (Reed et al., 2018). Our investigation revealed that self-rated health and self-management abilities exhibited spurious correlations or acted as indirect influencing factors in single-factor analysis. Thus, the spurious correlation was eliminated by accounting for the impact of specific variables (such as age, education, and IC) in a multi-factor analysis. Self-rated health can predict an individual’s future health status or self-management needs. Even two people living with the same disease or functional impairment may evaluate their health differently, and the source of this difference may be due to differences in their social determinants (Balaj, 2022). In multivariate linear regression analyses, the association between self-rated health and self-management abilities may have disappeared when social determinants were included.

Moreover, our study found no correlation between eHealth literacy and self-management in older adults with reduced IC. Previous studies have shown that eHealth literacy was a significant predictor of self-management behaviors and that older adults’ online engagement behaviors, such as through email or online social networking, were effective in improving older adults’ self-management (Scheffer et al., 2021; Wong et al., 2022), which is inconsistent with the findings of this study. The generic version of the eHealth Literacy Scale used in this study may not capture the eHealth literacy of the study population, given the social nature of Web 2.0 (Lee et al., 2021). Future studies should consider using appropriate eHealth literacy assessment tools to explore the correlation between eHealth literacy and self-management abilities.

While our study provides valuable insights, it is not without limitations. First, this study relied on self-reported data, which had a propensity to be influenced by personal opinions and perspectives. Second, since the sample was not selected by randomization, it is unlikely that the sample included was fully representative of the population being studied. This weakened the ability to generalize from the sample to the population of interest. Third, the study population was from the same county, which may prevent the results from being generalized nationwide and introduce sociodemographic selection bias. Finally, the cross-sectional design of this study did not permit causal inferences.

## 5 Conclusion

This study investigated IC and self-management ability status in older adults experiencing IC decline and identified the factors influencing self-management ability. We found a high prevalence of concurrent declines across the three dimensions of IC in this population. The self-management ability score of older adults experiencing IC decline was 67.05 ± 12.53 out of 100, mainly related to age, perceived social support, and IC composite scores, providing baseline information for healthcare managers and policymakers, underlining IC decline as a public health problem.

While the associations observed in this study suggest potential pathways for intervention, it is essential to note that these findings do not imply a causal relationship. Therefore, fostering self-management abilities through social support and mental health interventions may be beneficial for individuals living with IC decline, but further research is needed to establish effective strategies. To enhance the capacity of older adults to manage their health within the community, specialized training should be provided to community nurses responsible for aged care. In addition, an effective self-management program should be developed to assist older adults experiencing IC decline understand the aging process, address treatment-related challenges, and recognize how self-management abilities and social support can help manage the condition.

## Data Availability

The raw data supporting the conclusions of this article will be made available by the authors, without undue reservation.

## References

[ref1] Balaj M. (2022). Self-reported health and the social body. Soc. Theory Health 20, 71–89. doi: 10.1057/s41285-020-00150-0

[ref2] Belloni G. Cesari M. (2019). Frailty and intrinsic capacity: two distinct but related constructs. Front. Med. 6:133. doi: 10.3389/fmed.2019.00133, PMID: 31275941 PMC6591451

[ref3] Bos-Touwen I. Schuurmans M. Monninkhof E. M. Korpershoek Y. Spruit-Bentvelzen L. Ertugrul-van der Graaf I.. (2015). Patient and disease characteristics associated with activation for self-management in patients with diabetes, chronic obstructive pulmonary disease, chronic heart failure and chronic renal disease: a cross-sectional survey study. PLoS One 10:e0126400. doi: 10.1371/journal.pone.0126400, PMID: 25950517 PMC4423990

[ref4] Cesari M. Araujo de Carvalho I. Amuthavalli Thiyagarajan J. Cooper C. Martin F. Reginster J.. (2018). Evidence for the domains supporting the construct of intrinsic capacity. J. Gerontol. A Biol. Sci. Med. Sci. 73, 1653–1660. doi: 10.1093/gerona/gly011, PMID: 29408961

[ref5] Charlson M. E. Pompei P. Ales K. L. MacKenzie C. R. (1987). A new method of classifying prognostic comorbidity in longitudinal studies: development and validation. J. Chronic Dis. 40, 373–383. doi: 10.1016/0021-9681(87)90171-8, PMID: 3558716

[ref6] Chen H. Wang B. Lv R. Zhou T. Shen J. Song H.. (2023). Progression and trajectory network of age-related functional impairments and their combined associations with mortality. iScience. 26:108368. doi: 10.1016/j.isci.2023.108368, PMID: 38058300 PMC10696261

[ref7] Convery E. Hickson L. Meyer C. Keidser G. (2019). Predictors of hearing loss self-management in older adults. Disabil. Rehabil. 41, 2026–2035. doi: 10.1080/09638288.2018.1457091, PMID: 29587551

[ref8] Cui Y. Guo Z. Yang T. Liu Q. Liu N. Yang H.. (2023). Network analysis of negative emotion and self-management in Chinese patients with early chronic kidney disease. Curr. Psychol. 43, 10237–10246. doi: 10.1007/s12144-023-05111-0

[ref9] Feeney B. C. Collins N. L. (2015). A new look at social support: a theoretical perspective on thriving through relationships. Personal. Soc. Psychol. Rev. 19, 113–147. doi: 10.1177/1088868314544222 PMC5480897 25125368

[ref10] Folstein M. Folstein S. McHugh P. (1975). “Mini-mental state”. A practical method for grading the cognitive state of patients for the clinician. J. Psychiatr. Res. 12, 189–198. doi: 10.1016/0022-3956(75)90026-6 1202204

[ref11] Garnett A. Ploeg J. Markle-Reid M. Strachan P. H. (2018). Self-Management of Multiple Chronic Conditions by community-dwelling older adults: a concept analysis. SAGE Open Nurs. 4:2377960817752471. doi: 10.1177/2377960817752471, PMID: 33415188 PMC7774451

[ref12] Geboers B. de Winter A. F. Spoorenberg S. L. W. Wynia K. Reijneveld S. A. (2016). The association between health literacy and self-management abilities in adults aged 75 and older, and its moderators. Qual. Life Res. 25, 2869–2877. doi: 10.1007/s11136-016-1298-2, PMID: 27101999 PMC5065597

[ref13] George P. P. Lun P. Ong S. P. Lim W. S. (2021). A rapid review of the measurement of intrinsic capacity in older adults. J. Nutr. Health Aging 25, 774–782. doi: 10.1007/s12603-021-1622-6, PMID: 34179933 PMC7966899

[ref14] González-Bautista E. de Souto B. P. Andrieu S. Rolland Y. Vellas B. (2021). Screening for intrinsic capacity impairments as markers of increased risk of frailty and disability in the context of integrated care for older people: secondary analysis of MAPT. Maturitas 150, 1–6. doi: 10.1016/j.maturitas.2021.05.011, PMID: 34274071

[ref15] Guralnik J. M. Simonsick E. M. Ferrucci L. Glynn R. J. Berkman L. F. Blazer D. G.. (1994). A short physical performance battery assessing lower extremity function: association with self-reported disability and prediction of mortality and nursing home admission. J. Gerontol. 49, M85–M94. doi: 10.1093/geronj/49.2.M85, PMID: 8126356

[ref16] Jiang X. Chen F. H. Yang X. X. Yang M. Zhang X. H. Ma X.. (2023). Effects of personal and health characteristics on the intrinsic capacity of older adults in the community: a cross-sectional study using the healthy aging framework. BMC Geriatr. 23:643. doi: 10.1186/s12877-023-04362-7 37817083 PMC10566030

[ref17] Kim M. J. Bronas U. G. Quinn L. Sharp L. K. Park C. Gruss V.. (2023). Cognitive function and self-management behaviors in older adults with type 2 diabetes. Nurs. Res. 72, 38–48. doi: 10.1097/NNR.0000000000000624, PMID: 36097261

[ref18] Kim H. Sereika S. M. Albert S. M. Bender C. M. Lingler J. H. (2022). Do perceptions of cognitive changes matter in self-management behaviors among persons with mild cognitive impairment? Gerontologist 62, 577–588. doi: 10.1093/geront/gnab129, PMID: 34447996 PMC9019648

[ref19] Kroenke K. Spitzer R. L. (2002). The PHQ-9: a new depression diagnostic and severity measure. Psychiatr. Ann. 32, 509–515. doi: 10.3928/0048-5713-20020901-06

[ref20] Lan X. Lu X. Yi B. Chen X. Jin S. (2021). Factors associated with self-management behaviors of patients with chronic obstructive pulmonary disease. Jpn. J. Nurs. Sci. 19:2450. doi: 10.1111/jjns.12450 34398525

[ref21] Laursen D. H. Christensen K. B. Christensen U. Frølich A. (2016). Self-rated health as a predictor of outcomes of type 2 diabetes patient education programmes in Denmark. Public Health 139, 170–177. doi: 10.1016/j.puhe.2016.06.018, PMID: 27475450

[ref22] Lee J. Lee E. H. Chae D. (2021). eHealth literacy instruments: systematic review of measurement properties. J. Med. Internet Res. 23:e30644. doi: 10.2196/30644, PMID: 34779781 PMC8663713

[ref23] Leroi I. Simkin Z. Hooper E. Wolski L. Abrams H. Armitage C. J.. (2020). Impact of an intervention to support hearing and vision in dementia: the SENSE-cog field trial. Int. J. Geriatr. Psychiatry 35, 348–357. doi: 10.1002/gps.5231, PMID: 31713262 PMC7079053

[ref24] Leung A. Y. M. Su J. J. Lee E. S. H. Fung J. T. S. Molassiotis A. (2022). Intrinsic capacity of older people in the community using WHO integrated Care for Older People (ICOPE) framework: a cross-sectional study. BMC Geriatr. 22:304. doi: 10.1186/s12877-022-02980-1 35395736 PMC8993034

[ref25] Lex A. Gehlenborg N. Strobelt H. Vuillemot R. Pfister H. (2014). UpSet: visualization of intersecting sets. IEEE Trans. Vis. Comput. Graph. 20, 1983–1992. doi: 10.1109/TVCG.2014.2346248, PMID: 26356912 PMC4720993

[ref26] Liu Y. X. Du Q. F. Jiang Y. L. (2023). Detection rate of decreased intrinsic capacity of older adults: a systematic review and meta-analysis. Aging Clin. Exp. Res. 35, 2009–2017. doi: 10.1007/s40520-023-02515-7, PMID: 37543528

[ref27] Liu S. Kang L. Liu X. Zhao S. Wang X. Li J.. (2021). Trajectory and correlation of intrinsic capacity and frailty in a Beijing elderly community. Front. Med. 8:8. doi: 10.3389/fmed.2021.751586 PMC8695757 34957141

[ref28] Lu R. Li Y. Zheng Z. Yan Z. (2021). Exploring factors associated with self-management compliance among rural elders with diabetes. Inquiry 58:469580211012491. doi: 10.1177/00469580211012491, PMID: 33899547 PMC8082998

[ref29] Lundberg P. C. Thrakul S. (2012). Type 2 diabetes: how do Thai Buddhist people with diabetes practise self-management? J. Adv. Nurs. 68, 550–558. doi: 10.1111/j.1365-2648.2011.05756.x, PMID: 21711465

[ref30] Luo X. Liu T. Yuan X. Ge S. Yang J. Li C.. (2015). Factors influencing self-Management in Chinese Adults with type 2 diabetes: a systematic review and Meta-analysis. Int. J. Environ. Res. Public Health 12, 11304–11327. doi: 10.3390/ijerph120911304 26378555 PMC4586677

[ref31] Manohar N. MacMillan F. Steiner G. Z. Arora A. (2019). “Recruitment of research participants,’’ in Handbook of research methods in health social sciences. ed. P. Liamputtong (Singapore: Springer).

[ref32] Prince M. J. Acosta D. Guerra M. Huang Y. Jacob K. S. Jimenez-Velazquez I. Z.. (2021). Intrinsic capacity and its associations with incident dependence and mortality in 10/66 dementia research group studies in Latin America, India, and China: a population-based cohort study. PLoS Med. 18:3097. doi: 10.1371/journal.pmed.1003097 PMC8439485 34520466

[ref33] Quinn C. Toms G. Jones C. Brand A. Edwards R. T. Sanders F.. (2016). A pilot randomized controlled trial of a self-management group intervention for people with early-stage dementia (the SMART study). Int. Psychogeriatr. 28, 787–800. doi: 10.1017/S1041610215002094, PMID: 26674087

[ref34] Rarajam Rao A. Waris M. Saini M. Thakral M. Hegde K. Bhagwasia M.. (2023). Prevalence and factors associated with impairment in intrinsic capacity among community-dwelling older adults: an observational study from South India. Curr Gerontol Geriatr Res. 2023, 1–9. doi: 10.1155/2023/4386415 PMC10148741 37128497

[ref35] Reed R. L. Roeger L. Howard S. Oliver-Baxter J. M. Battersby M. W. Bond M.. (2018). A self-management support program for older Australians with multiple chronic conditions: a randomised controlled trial. Med. J. Aust. 208, 69–74. doi: 10.5694/mja17.00127 29385967

[ref36] Rubenstein L. Z. Harker J. O. Salvà A. Guigoz Y. Vellas B. (2001). Screening for undernutrition in geriatric practice: developing the short-form mini-nutritional assessment (MNA-SF). J. Gerontol. A Biol. Sci. Med. Sci. 56, M366–M372. doi: 10.1093/gerona/56.6.M366, PMID: 11382797

[ref37] Scheffer M. M. J. Menting J. Boeije H. R. (2021). Self-management of social well-being in a cross-sectional study among community-dwelling older adults: the added value of digital participation. BMC Geriatr. 21:539. doi: 10.1186/s12877-021-02482-6 34635080 PMC8504001

[ref38] Schulman-Green D. Jaser S. S. Park C. Whittemore R. (2016). A meta-synthesis of factors affecting self-management of chronic illness. J. Adv. Nurs. 72, 1469–1489. doi: 10.1111/jan.12902, PMID: 26781649 PMC4891247

[ref39] Schuurmans H. Steverink N. Frieswijk N. Buunk B. P. Slaets J. P. Lindenberg S. (2005). How to measure self-management abilities in older people by self-report. The development of the SMAS-30. Qual. Life Res. 14, 2215–2228. doi: 10.1007/s11136-005-8166-9, PMID: 16328901

[ref40] Shrestha M. Ng A. H. Gray R. J. (2021). Association between subthreshold depression and self-care behaviours in adults with type 2 diabetes: a protocol for a cross-sectional study. J. Clin. Nurs. 30, 2453–2461. doi: 10.1111/jocn.15250, PMID: 32415880

[ref41] Skolasky R. L. Green A. F. Scharfstein D. Boult C. Reider L. Wegener S. T. (2011). Psychometric properties of the patient activation measure among multimorbid older adults. Health Serv. Res. 46, 457–478. doi: 10.1111/j.1475-6773.2010.01210.x, PMID: 21091470 PMC3064914

[ref42] Steptoe A. Deaton A. Stone A. A. (2015). Subjective well-being, health, and ageing. Lancet 385, 640–648. doi: 10.1016/S0140-6736(13)61489-0, PMID: 25468152 PMC4339610

[ref43] Suglo J. N. Evans C. (2020). Factors influencing self-management in relation to type 2 diabetes in Africa: a qualitative systematic review. PLoS One 15:e0240938. doi: 10.1371/journal.pone.0240938, PMID: 33091039 PMC7580976

[ref44] Tangalos E. G. Smith G. E. Ivnik R. J. Petersen R. C. Kokmen E. Kurland L. T.. (1996). The Mini-mental state examination in general medical practice: clinical utility and acceptance. Mayo Clin. Proc. 71, 829–837. doi: 10.4065/71.9.829 8790257

[ref45] Tavassoli N. Piau A. Berbon C. De Kerimel J. Lafont C. De Souto B. P.. (2021). Framework implementation of the INSPIRE ICOPE-CARE program in collaboration with the World Health Organization (WHO) in the Occitania region. J. Frailty Aging 10, 103–109. doi: 10.14283/jfa.2020.26, PMID: 33575698

[ref46] van Schie D. Castelein S. van der Bijl J. Meijburg R. Stringer B. van Meijel B. (2016). Systematic review of self-management in patients with schizophrenia: psychometric assessment of tools, levels of self-management and associated factors. J. Adv. Nurs. 72, 2598–2611. doi: 10.1111/jan.13023 27200500

[ref47] Ventry I. M. Weinstein B. E. (1983). Identification of elderly people with hearing problems. ASHA 25, 37–42, PMID: 6626295

[ref48] von Elm E. Altman D. G. Egger M. Pocock S. J. Gøtzsche P. C. Vandenbroucke J. P. (2014). The strengthening the reporting of observational studies in epidemiology (STROBE) statement: guidelines for reporting observational studies. Int. J. Surg. 12, 1495–1499. doi: 10.1016/j.ijsu.2014.07.013 25046131

[ref500] World Health Organization. (2017). Integrated care for older people (ICOPE): guidelines on community-level interventions to manage declines in intrinsic capacity. World Health Organization. Available at: https://iris.who.int/handle/10665/341989. 29608259

[ref50] WHO Guidelines Approved by the Guidelines Review Committee (2017). Integrated Care for Older People: guidelines on community-level interventions to manage declines in intrinsic capacity. Geneva: World Health Organization. 29608259

[ref51] Wong A. K. C. Bayuo J. Wong F. K. Y. (2022). Investigating predictors of self-care behavior among homebound older adults: the role of self-efficacy, eHealth literacy, and perceived social support. J. Nurs. Scholarsh. 54, 278–285. doi: 10.1111/jnu.12730, PMID: 34766694

[ref52] World Health Organization (2015). World report on ageing and health. Geneva: World Health Organization.

[ref53] Xu R. Zhou L. Lu S. Wong E. Chang J. Wang D. (2020). Psychometric validation and cultural adaptation of the simplified Chinese eHealth literacy scale: cross-sectional study. J. Med. Internet Res. 22:e18613. doi: 10.2196/18613, PMID: 33284123 PMC7752540

[ref54] Zeng X. Shen S. Xu L. Wang Y. Yang Y. Chen L.. (2021). The impact of intrinsic capacity on adverse outcomes in older hospitalized patients: a one-year follow-up study. Gerontology 67, 267–275. doi: 10.1159/000512794, PMID: 33735899

[ref55] Zimet G. D. Dahlem N. W. Zimet S. G. (1988). The multidimensional scale of perceived social support: journal of personality assessment. J. Pers. Assess. 52, 30–41. doi: 10.1207/s15327752jpa5201_2

[ref56] Ziv I. Caspi D. CoJocaru D. (2024). Self-management elucidates how practicing physical exercises influences the health related quality of life of independently dwelling older adults. Inquiry 61:00469580241271272. doi: 10.1177/00469580241271272, PMID: 39323068 PMC11440539

